# Two Sides of the Same Coin: How to Integrate Social Network Analysis and Topic Detection to Investigate Shared Contents and Communicative Interactions in Social Representations

**DOI:** 10.5334/irsp.973

**Published:** 2024-12-16

**Authors:** Valentina Rizzoli, Anderson da Silveira, Mirella De Falco, Mauro Sarrica

**Affiliations:** 1 Department of Communication and Social Research, Sapienza University of Rome, IT; 2 Department of Psychology, Federal University of Santa Catarina, BR

**Keywords:** social representations, social network analysis, community detection, topic detection, Reinert method, groups

## Abstract

This paper advances the integration of Social Network Analysis (SNA) and topic detection into the study of Social Representations (SRs). We suggest that a combination of the two analyses helps to detect communities characterised by shared contents and/or social interactions, the two facets that make representations ‘social’. Building on Moliner’s (2023) proposal we present a step-by-step approach to combine the identification of shared meanings based on lexicometric analysis and identification of social interaction based on social network analysis techniques. To illustrate our proposal, we use a dataset of 396 Brazilian tweets about the Covid-19 pandemic that was collected to investigate the SR of science during the pandemic. The Reinert method was run on the corpus using the Iramuteq R interface and a bipartite network analysis was performed using Gephi software. We thus operationalised 615 users and six topics as nodes, while shared topics and interactions (883 mentions) as arcs. This allowed us to examine both the content of social representations and interactions among different individuals and communities. In our case, the results highlight shared content as the main determinant for community formation; however, some users appear to have linked different communities together: they are associated to a community not because of the topic they share, but because of their interactions with other users. We contend this methodology proves to be a fruitful theoretical-methodological link between SNA and SR theory, as it detects both facets of the relationship between SRs and groups: the shared contents and the communicative interactions between individuals.

Social representations (SRs; Moscovici in 1961/1976) refer to dynamic systems of beliefs, practices, values, opinions, and attitudes related to a social object that are disseminated, negotiated, and transformed through social interactions (Jodelet 1989). Through SRs, people and groups co-create common-sense theories that guide their interpretation and discussion of the object in question. From this definition, several facets of the relationship between groups and social representations can be derived: SRs emerge through the interaction and communication among members of social groups; group membership may affect exposure to, acceptance of, and use of SR; once established, groups compete in the social arena to foster SRs of objects which are relevant to them or that serve intragroup and intergroup interests; groups may be the object of representation, either directly or indirectly (e.g., when outgroups are representational objects). From a communicative perspective, SRs define group boundaries by enabling communication among members and delineating those who share similar denotations and connotations of the representational object. Simultaneously, individuals – far from being fixed entities – draw on different organising principles of SRs depending on their discursive positioning in social networks. Howarth (2001) extends this argument by arguing that SRs not only shape multiple versions of group identities but can be manipulated to marginalize and exclude other groups or used to challenge existing social realities. Finally, as noted by Breakwell (1993), intragroup and intergroup dynamics (e.g., power relationships among groups and individuals) affect not only the content of SRs but also their structure and form, allowing for more dispersed or more cohesive forms of representation depending on the degree to which groups are structured.

From this perspective, similarities can be observed between the concept of ‘group’ in Social Representation Theory (SRT) and the notion of ‘community’ in communication sciences, particularly in the study of social networks. The classic idea of ‘community’ in communication sciences is now better understood as fluid social networks rather than geographically bound entities (Willson 2010). Online social networks contribute to the formation of communities, no longer tied to a common physical location but to virtual environments hosted on digital platforms such as Facebook, Twitter (now X), Instagram, and others (Recuero et al. 2020). These online environments provide real-time communication resources, enabling people to share opinions, beliefs, and form connections with the ‘offline world’ (Santaella & Lemos 2010). Thus, from this viewpoint, a community is defined by the ties among its members, whether based on shared ideas or common spaces – physical or virtual.

Within SRT, multiple approaches have been developed to explore the above-mentioned facets of group structure and dynamics. For instance, the relationship between cognitive system and normative metasystem, the position of individual in such metasystem, and the role of SRs in organising the symbolic relationships between individual and groups is at the core of the socio-dynamic approach and of its translation into societal psychology (Doise et al. 1993; Doise 1990). Identity Process Theory, introduced by Breakwell (1993), highlights several identity functions that are served by SRs and vice versa: changes in group memberships, group dynamics, or value shifts necessitate a re-evaluation of identity elements, reflecting the ongoing interplay between identity and SRs. Indeed, negotiation and transformation are inherent in the adoption of SRs (Voelklein & Howarth 2005), with group boundaries being fluid and yet extremely important for defining the group identity. Finally, to avoid the risk of reducing groups to aggregates of psychological characteristics, and to consider homogeneity among individuals’ cognitions as overlapping to representational processes, Wagner suggests distinguishing between *nominal* and *reflexive* groups. Whereas the former, like aggregates, can be defined from the outside according to some external criterium, it is in the latter that members are aware of the group as a meaningful unit, and actively contribute to the negotiation and maintenance of shared meaning among members and in relationship with the normative metasystem. Together with Wachelke (2012) we suggest that in order for a group to be considered a knowledge system, it must be a reflexive group: sharing SRs is a matter of being able to adopt a reflexive posture, therefore regulating intragroup and intergroup relations while engaging in meaning making processes. Wagner (1995) emphasises this as the ‘holomorphic’ characteristic of SRs: in order to be meaningful and in any case to have a inter-relationship with ‘others’, even idiosyncratic positions must refer to a shared frame of reference, that contains meta-information about normative regulations and broader networks of signification, and that serves to (de)legitimize the actor point of view.

These assumptions among the relationship between SRs and groups inspired diverse methods and techniques aimed at identifying what is shared and by whom. A long tradition of studies rooted in SRT focuses on textual data, and with the diffusion of software studies have increasingly employed one or more techniques to quantitatively analyse textual data. Researchers, for example, run correspondence analysis to examine the co-occurrence of words collected via free association tasks (Doise, et al. 1993), or employ more complex algorithm to examine semantic clusters emerging from large textual corpora (e.g., Reinert 1986), or to find the central core components of the representational structure (Aim et al. 2018), or the representational content along time (Rizzoli et al. 2017), or to study the intertwining of social representations, attitudes, and identities (Rizzoli et al. 2024a), or apply graphs theory through similarity analysis to examine lexical areas around key terms (Degenne & Vergès 1973).

However, when dealing with textual corpora, research often focuses on association between contents (e.g., free associations, words extracted from texts), and nominal groups (e.g., groups defined on the basis of sociological or psychosocial categorization). The connection between communicative processes, broader network of signification, and group dynamics in defining SRs contents and forms are often overlooked. This is particularly surprising given that, over the past two decades, the rise of the internet and social media has allowed researchers to document social interactions on an unprecedented scale.

One of the reasons for the lack of research addressing the intersection between SRs, groups and online communicative processes, is the need to better define ways in which online communities can be operationalized and studied within this specific field. To answer this challenge, Moliner (2023) suggests that Social Network Analysis (SNA) could be employed. Despite indicating that the notion of network analysis is not something new in SRT, the author points out that:

We can thus see that SRs have been considered as networks for several decades. However, the methods that have been developed for this purpose have never really drawn all the consequences of the premises on which they were based. Indeed, if we consider a SR as a network of opinions or beliefs, this network must include the individuals who have expressed these opinions or beliefs. Neither similarity analysis nor methods inspired by the study of social networks do this. This prevents these methods from identifying subgroups of individuals. This is why we suggest a different approach (Moliner 2023, p. 9).

To the question ‘How then can subgroups be identified in a supposedly homogeneous population?’ (Moliner 2023, p. 7), the author answers that the use of SNA in SRT requires approaches that not only locate the relationships between the opinions and beliefs of the participants, but also identify the relationships that exist between the participants.

Following and expanding on Moliner’s proposal, we suggest that: a) adopting discrete cognitive elements as units of analysis – such as words – is only one aspect of the representational structure, while shared meanings can be defined more widely by looking at broader lexical worlds, often referred to as ‘topics’; b) SNA, especially when applied to data collected online via media platform, can help us in jointly considering topics (shared ideas) and the virtual shared space (interaction dynamics).

In sum, we aim to expand on Moliner’s idea, combining bipartite structural network analysis with topic detection analysis to capture both the content of social representations and the value derived from group members’ interaction and communication. To empirically test these ideas, we analyse data from Twitter (X),^1^ a microblogging platform where users post messages, mention others, repost content, or comment on third-party posts.

In the next paragraphs after a very brief introduction to topic detection methods of analysis which are well known in the SR field, we deepen some key concepts of SNA. We will then use Twitter (X) data to exemplify how a combination of topic detection and SNA could provide an analytical approach for the comprehensive understanding of community detection in the analysis of SRs.

## Topic Detection and Social Representations

Topic detection can be defined as a procedure that allows summarising the content of a corpus through the identification of thematic clusters of words. Several methods of analysis have been developed to achieve the goal of extracting essential information, obtaining an overview of the content, and consequently classifying texts automatically and in an unsupervised manner (i.e., without a pre-defined classification system). These methods can be mainly categorised into two approaches (Sbalchiero 2018): one developed within the realm of computer science, which is machine learning-based and includes topic models such as Latent Dirichlet Allocation (Blei et al. 2003) and the subsequent tradition of probabilistic topic models (e.g., Structural Topic Model, Roberts et al. 2019; Correlated Topic Model, Blei & Lafferty 2007). The other approach is derived from the French school of data analysis (Benzécri 1973) to perform content analysis automatically within the realm of the social sciences.

In the current paper, we will adopt the second approach, relying on one of the most known analyses within it, which is the Reinert method (1993; 1986). It has been widely adopted in SRs research to identify shared understandings of objects and emerging topics in the societal arena (e.g., Chartier & Meunier 2011; Emiliani et al. 2020; Lahlou 1996; Lahlou 2011; Rizzoli et al. 2024b; Sarrica et al. 2020). The Reinert method allows for analysing the structure of a corpus by examining word co-occurrences within text segments and identifying clusters of words representing a class of meaning (i.e., topics). These clusters, which are defined by Reinert (1998) lexical worlds, are considered as visible traces (lexical) of the latent dimensions that underlie the discourse and are often interpreted as topics expressed through recurring lexical patterns in discourse. Thanks to the adoption of softwares – such as Alceste or Iramuteq (Ratinaud 2009) – it is possible to systematically identify these patterns by detecting elementary context units (ECUs; i.e., text segments) from initial context units (ICU; i.e., texts) and constructing a contingency matrix of word x ECU co-occurrences. Hierarchical clustering is then applied to group ECUs with similar lexical profiles into distinct classes, forming lexical worlds/topics. The resulting dendrogram illustrates the hierarchical organisation of the topics. The procedure allows to identify keywords and text segments (ECU) that best represent each topic, based on χ2 metric.

## Social Network Analysis in the Social Sciences

Despite their importance, topic detection methods often overlook the role of interaction and communicative processes in SRs, an issue that can be addressed through SNA. Rooted in structural sociology, SNA dates back to early 20th-century theorists like Simmel and Moreno, who emphasised social interactions in shaping perceptions. During the 1930s, Kurt Lewin proposed a topological approach to group interaction and at the same time principles of Gestalt Theory were included into the novel science of ‘sociometry’ by Moreno (1934). Moreno’s 1930s work in sociometry laid the foundation for mapping human relationships using sociograms to identify central and isolated actors, a practice still common today (Scott 2012; 2017).

SNA advanced in the 1970s with Granovetter’s (1973) work on weak-tie networks, key to information dissemination and social mobility. Recent techniques now allow structured visualisation of social interactions, revealing previously hidden patterns (Hansen et al. 2020). However, as Moliner (2023) points out, SNA remains underutilized in social psychology, particularly within SRT, despite its potential for theoretical integration.

Modern SNA has four defining traits (Freeman 2004; Prell & Schaefer 2024):

1. it focuses on the ties linking social actors,
2. it is grounded in systematic empirical data,
3. it uses graphic imagery, and
4. it applies mathematical and computational models.

Sociometry and structural sociology provided SNA’s ‘sociological basis’ while graph theory offered the mathematical tools for analysing networks (Recuero et al. 2020). Key explanatory theories include Granovetter’s strong and weak ties (1983), Burt’s structural holes (1992), Lemieux’s groupality (1993), and relationship coordination (1998). All are relational theories, emphasising the characteristics of relationships between actors rather than individual attributes.

### Network Elements

Network elements^2^ are essential to SNA, which measures the relationships between network nodes. Networks consist of entities (nodes) and their connections. Entities can represent individuals, groups, or institutions. Networks may be unimodal (nodes represent the same entity) or bimodal/bipartite (nodes represent different entities, like individuals and places) (Prell & Schaefer 2024). This paper explores how bimodal networks can be applied to the study of SRs, enabling the identification of relationships between individuals (through communicative exchange) and between individuals and topics (shared representational contents or ideas).

**Figure 1:**
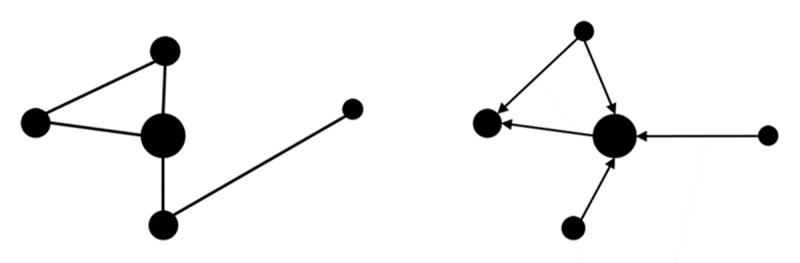
Non-directed network (left) and directed network (right).

The relationships between two points can express different types of connections, such as associations or citations in a social media. Connections between nodes can be non-directed (edges) or directed (arcs) (Figure 1; e.g., Lemieux & Ouimet 2012; Prell & Schaefer 2024). Various metrics assess node importance, including degree centrality (number of direct connections), betweenness (nodes acting as intermediaries), and eigenvector centrality (influence of a node based on its connections) (Lemieux & Ouimet 2012; Recuero et al. 2020).

In terms of communities, Recuero (2009) explains that social networks typically form clusters where nodes within the community exchange information more frequently than with external nodes. While offline networks rely on territorial proximity, online networks are deterritorialized, forming connections through shared interests. These networks can have different topologies – association, affiliation, or emergent – based on direct relationships or dynamic conversations around specific topics. Emergent networks, in particular, are driven by the exchange of opinions on shared issues, and modularity algorithms are often used to detect these clusters or communities (Evkoski et al. 2021).

Connection metrics, such as the strength and valence of relationships, indicate the intensity and nature of interactions. Network-wide metrics like density and modularity measure overall connectivity and identify subgroups. Modularity is especially relevant for identifying groups as defined in SRT (often referred to as ‘communities’ in SNA), as dividing the network into groups/communities can reveal contexts where different opinions, attitudes, and images about a certain object circulate. Specialized software, such as UCINET, Gephi, and NodeXL, facilitates these analyses, though researchers must still interpret metrics in the context of their specific study goals.

## Social Network Analysis in Social Representations

In SRT, network analysis has primarily been used to analyse semantic content through Semantic Network Analysis. However, few studies have combined SNA to explore the relationship between group structure and SR production. A brief review identified studies by Muncer et al. (1996), Laroche and Plante (2022), Chen et al. (2022), and Moliner (2023).

Muncer et al. (1996) compared SRs of crime between American and British criminologists, using network analysis to reveal different representations of crime causes in each country, based on survey data. Laroche and Plante (2022) mapped actor networks related to emergency risk management in a community, using interviews to create one-mode and bipartite networks that linked actors to identified risks, showing how SRT can reveal social dynamics. Chen et al. (2022) analysed 40 million messages on the Chinese platform Weibo, using machine learning to track changing SRs of COVID-19 among 9.7 million users. They found strong associations between the SRs of the general public and opinion leaders, but little influence from public organisations’ SRs.

Moliner’s (2023) research is notable for integrating network analysis into the study of SRs, marking a significant theoretical-methodological connection between SNA and SRT. Moliner conducted three studies to explore methods for detecting heterogeneity or homogeneity within groups in SR research. He introduced a bipartite network analysis for analysing free association data, showing how associations can be linked to participants. This approach identifies sub-groups based on shared vocabularies (proxies for shared representational content) rather than predefined groups, demonstrating SNA’s relevance in assessing the homogeneity or heterogeneity of representations.

## Aim

Despite all the positive considerations about the use of SNA for the study of social representations, Moliner (2023) argues that there are still many uncertainties that require future research, these include the statistical criteria used in the algorithms for creating communities, consensus between verbal association tasks, the social identity of individuals, and the centrality of nodes (e.g., opinions, individuals) in networks.

Aim of the current paper is to contribute to this debate and to foster the use of SNA to study SRs. We propose a methodological approach jointly based on topic detection and SNA which we expect to provide a number of advantages:

- by using a topic-based approach, it does not rely solely on single words but on semantic universes, and builds on methods of analysis which have been largely applied within SRT to examine a variety of textual data (from free associations to large natural language corpora);
- by using a bipartite network, it simultaneously shows the relationship *users–topics* and the relationship *users–users*;
- by combining both topic detection and SNA it should identify bottom-up homogeneous as well as non-homogeneous groups and provide detailed measures of these connections, based on individual-topics (shared meanings) as well on their individual-individual (interaction and communication) connections, and not on previously defined categories.

In sum, we expect the method to detect two intertwined facets of SRs, and to examine two basic assumptions of the theory: the biunique relationship between shared contents and group boundaries, and the communicative interactions which are crucial for SRs microgenesis and sociogenesis.

**Figure 2:**
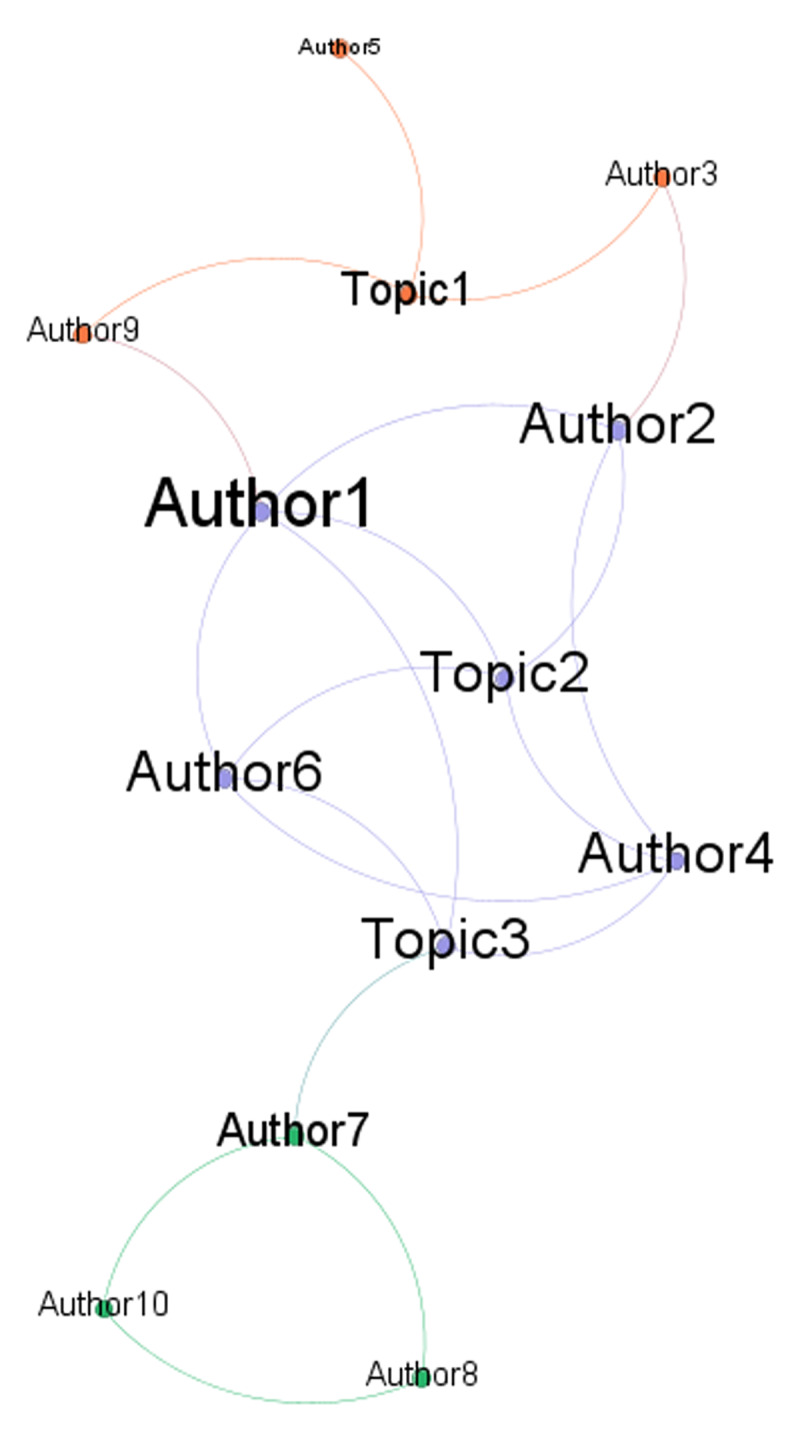
Ideal example of three different group formations performing a bipartite network analysis: a) the orange group exists because its members (i.e., authors 3, 5, and 9) share the same representational contents (i.e., Topic 1); b) the green group exists because its members (Authors 7, 8, and 10) interact; c) the purple group exists because its members (Authors 1, 2, 4, and 6) both share the same representational contents (Topic 2 and 3) and interact with each other. Note: Each colour represents a group based on modularity class. The dimension of the nodes’ labels is proportional to the in-degree.

In particular, we expect that this approach could identify three ideal-typical situations (Figure 2): a) a group/community defined by the fact that its members share the same topic (i.e., representational content; confirming the assumption of shared contents but not the one about interaction); b) a group defined because its members interact (i.e., communicate each-other; confirming the assumption of communicative interaction but not the one about shared contents); c) a group defined by shared contents and interaction (i.e., confirming both the assumptions concerning the social nature of social representation). Moreover, each of the elements of these ideal-typical situations could be further described through social network metrics.

## Example of Application

### Dataset and Analyses

To test the proposed analysis method^3^, we used a sample of 396 Brazilian tweets about the Covid-19 pandemic collected via the Twitter API Master.^4^ Twitter (X) enables users to share messages with multiple recipients simultaneously, allowing a single post to be reshared and amplified across different users. This structure supports public debate and interaction, making Twitter (X) an ideal platform for studying how SRs emerge and evolve through online communication. Users can post messages, mention other profiles, repost content, or comment on third-party posts. The platform personalizes content based on users’ contacts, trending topics, and interests. Since Twitter (X) functions as a network, user interactions (e.g., liking, commenting, or retweeting) affect not only their own timeline but also those of others (Recuero et al. 2020).

The data was collected in February 2023 and the data used in the example covers the period from April 2 to 7, 2020. Only complete messages containing the word ‘covid’ and ‘science’, published in Portuguese, were selected, using the expression ‘Ciência Covid Lang:pt’ as the search term. For each tweet, we maintained information about the author and any mentions (including replies).

We choose a relatively small corpus of 13,863 occurrences and 3,692 word-type to make the derived output more easily readable. In fact, a dataset composed of tweets will most likely turn out to have many users and thus many nodes.

First, we run the Reinert method on the corpus using the Iramuteq R interface (Ratinaud 2009). Each tweet was considered as an ECU (i.e., the unit of analysis within which calculate the co-occurrences to identify the topics). The Reinert method not only provides word clusters associated with topics, but also returns a classification of ECUs (the entire tweet in this case) within the topics. This means that, at the end of the analysis, it is possible to extract a data matrix where each tweet will be associated with a user and a topic (consequently each individual who posted can be associated with a topic), in addition to the initial information (e.g., mentions). Reinert’s method identified six topics, comprising 90.4% of the tweets (358 tweets out of 396 were classified). As a result, we had a matrix consisting of 358 tweets (the ones classified by the Reinert method) and the following characteristics (columns): the author of the tweet, any mentions, and the topic label (from 1 to 6).

The dataset was used to perform the bipartite network analysis using Gephi software (Bastian et al. 2009). Topic (n = 6) and users (n = 614^5^) were entered as nodes (cf. Nodes file in the supplementary material).

As regards the connections, the arcs consist of 1) each time a user is linked to a topic (i.e., a user is associated to a representational content); 2) each time a user mentions another one (i.e., two users interact) (cf. Arcs file in the supplementary material). Following Recuero’s (2009) definition of group identification, we analysed *emergent networks* (i.e., networks formed around conversations on specific topics) generated from discussions about science in relation to the Covid-19 pandemic.

As regards the metrics used to generate the groups thus constructing the output, the nodes have been organised by in-degree, and the size of the node labels is proportional to the in-degree. The network is directed, based on user-topic (who refers to a topic) and user-user (who mentions whom) relationship. In the case of the user-topic connection, the arc weight (i.e., visualised as thickness) is determined by the number of messages from a user that are associated with a given topic. In the case of user-user connection, the weight is determined by the number of mentions. In operational terms, the group is thus defined by the strength of the association between the content of the representation and the user sharing that content, as well as by how often a user has interacted through communication in the virtual space with another user. To detect the communities/group we use a modularity algorithm. There are several modularity algorithms that identify sets of nodes with a higher density of interactions that are closer and more connected to each other (Evkoski et al. 2021). Modularity is a widely used metric in Gephi for detecting communities within a network, assessing the number of distinct node clusters (Cherven 2015; Hansen et al. 2020). In this study, the modularity test was conducted using the algorithm integrated into the Gephi software. The test parameters included the randomisation of arcs and the use of arc weights, with a resolution of 1.0. However, modularity can be measured with plug-ins from different algorithms that can be installed in this software. Cherven (2015, p. 189) points out that despite the various parameters used, ‘the ultimate goal of any of these algorithms is to group nodes based on the strength of their relationships’.

### Results

**Figure 3:**
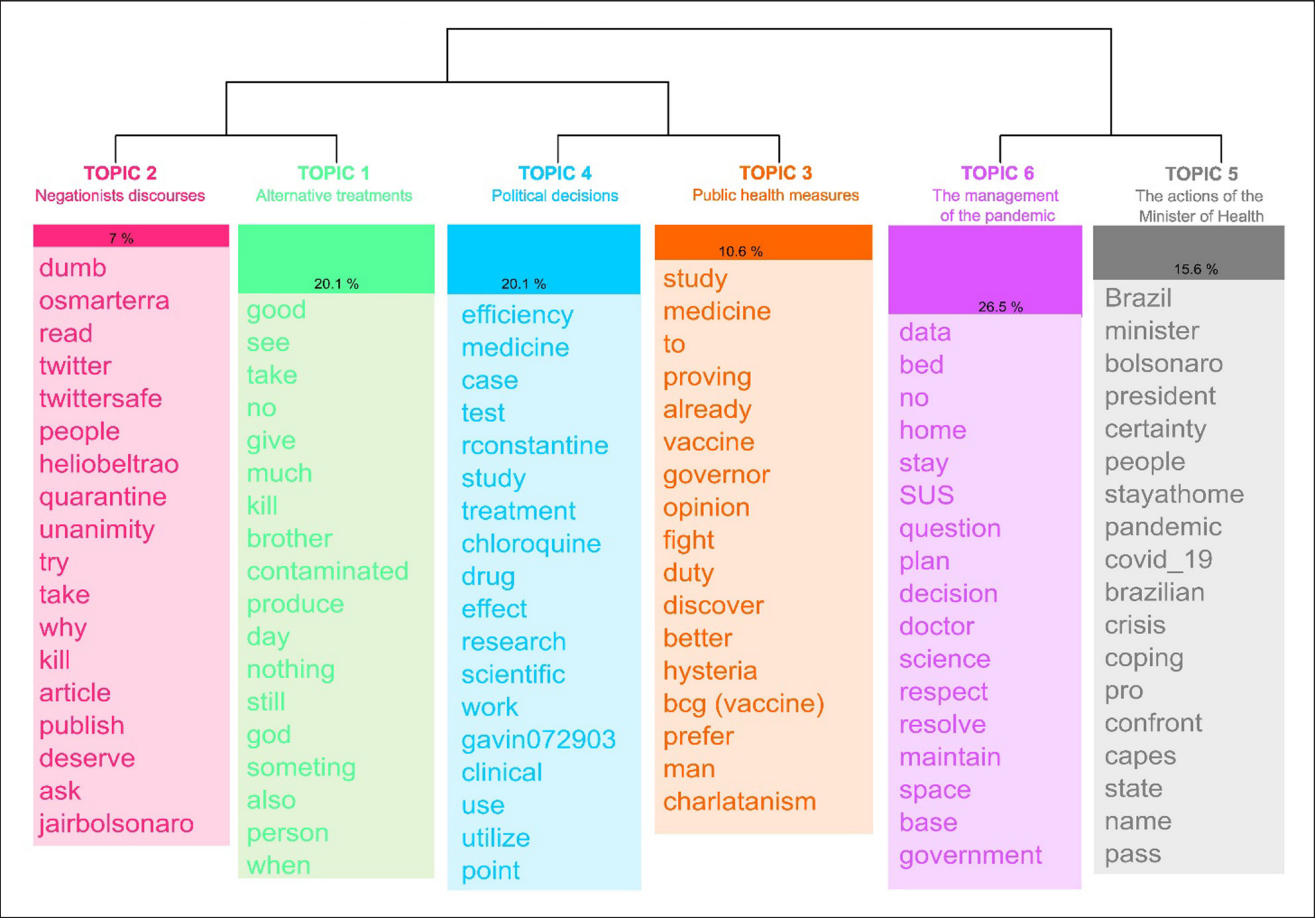
Dendrogram of the topics resulting from the Reinert method. English translation by the authors.

The topic detection identified six topics (Figure 3). Starting from a qualitative reading of the words associated with each topic, how they were used in the tweet, and the tweets associated with the topic, it was possible to assign a label to each topic.

From left to right, topics gather contents that refer to *Negationists discourses* (topic 2); *Alternative treatments* (e.g., *chloroquine*) (topic 1); *Political decisions* (topic 4); *Public health measures* (topic 3); *The management of the pandemic* (topic 6); *The actions of the Minister of Health* (topic 5). There is a heterogeneous debate in all the classes analysed, with polarised opinions. There is no consensus on the topics discussed in the corpus, but rather a conflict between opinions in favour of and against science, health measures and the use of drugs such as chloroquine. Another phenomenon observed is that the opinions expressed in the messages are validated on the basis of agreement or disagreement with political actors (e.g., @bolsonaro, @OsmarTerra, @jdoriajr, @lhmandetta) or journalists/media outlets (e.g., @monicabergamo, @FMouraBrasil, @folha). This will become more evident in the analysis of the bipartite network. People tend to mention these profiles to express agreement or disagreement, generating a conversation around controversial topics.

**Figure 4:**
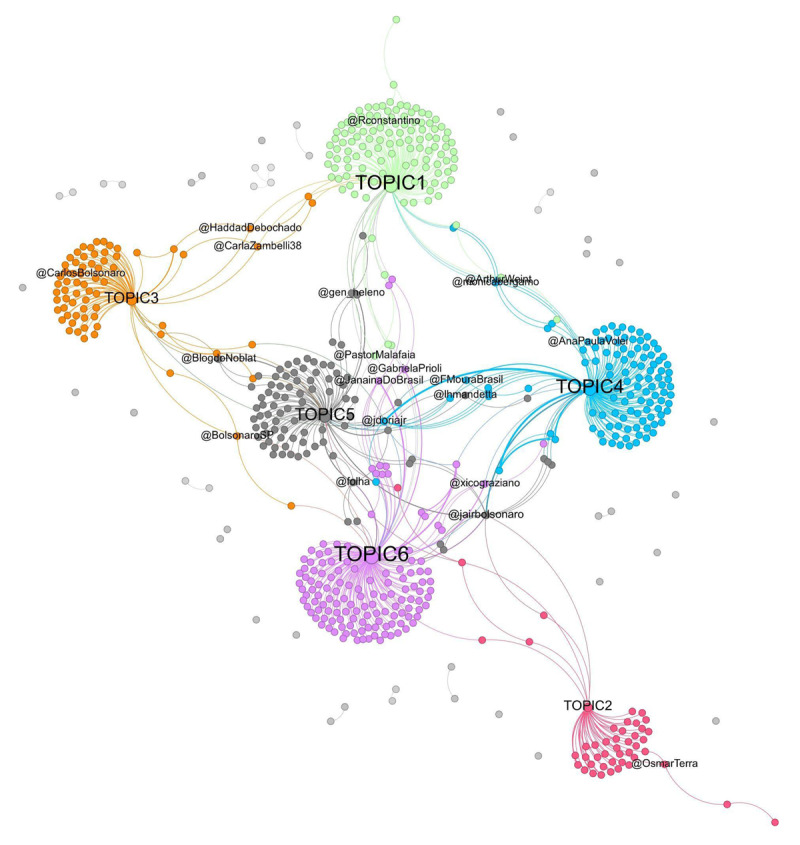
Bipartite network analysis output of the example of application (cf. graph file in the supplementary material). The thickness of the arc is determined by their weight. The size of the node labels is proportional to the in-degree. Each community is represented by a different colour and is defined by the modularity class.

As expected, the use of network analysis techniques allows us to investigate the picture in greater detail. Results identify six main groups/communities, mainly composed of individuals who share the same topic (Figure 4). For most individuals in the considered sample there is no direct interaction, so the main results fit with the first ideal-typical case: there is a shared content but apparently no explicit and direct interaction (of course we cannot exclude that the authors read each other).

By observing the ten profiles with the highest in-degree (i.e., those mentioned most; Table 1), we notice that five of them are associated with Brazilian politicians. Despite the small size of the dataset analysed, this information illustrates that, during the Covid-19 pandemic in Brazil, the debate about the disease was largely permeated by political discussions about which measures should be adopted, as shown in Figure 3. It is important to note that, in this context, former president Jair Bolsonaro and the former governor of São Paulo, João Doria (the two most mentioned profiles), stood out for their opposing views. While Bolsonaro opposed measures such as vaccination and lockdown, Doria defended these pandemic control strategies. These positions generated strong discussions and attacks on social media.

**Table 1:** List of profiles with the highest indegree in the network.


RANKING	ID	PROFILE	ACTIVITY	INDEGREE

1	308	@jairbolsonaro	President of Brazil	8.0

2	314	@jdoriajr	Governor of Sao Paulo	7.0

3	247	@folha	Newspaper	4.0

4	311	@JanainaDoBrasil	São Paulo State Representative	4.0

5	90	@BlogdoNoblat	Journalist	3.0

6	257	@GabrielaPrioli	Lawyer and Political Commentator	3.0

7	615	@XicoGraziano	Politician	3.0

8	260	@Gavin07290309	---	3.0

9	40	@AnaPaulaVolei	Political Commentator	2.0

10	61	@ArthurWeint	Minister of Education (Bolsonaro Government)	2.0


**Figure 5:**
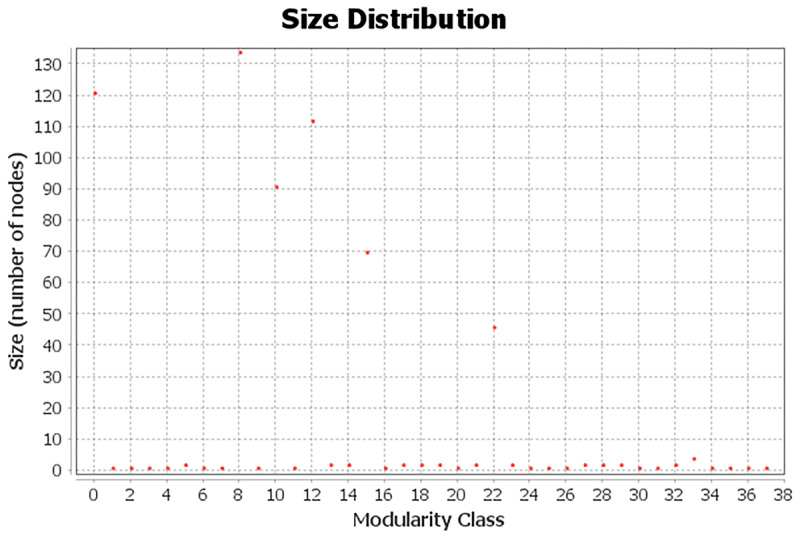
The figure shows the distribution of community sizes by modularity class, highlighting classes 0, 8, 10, 12, 15, and 22. These classes represent communities formed by nodes connected to topics 1 through 6, identified using the Reinert Method. Each of these nodes is linked to at least one of the six topics. Other modular groups, which are not related to these topics, only represent ‘user-user’ relationships.

To detect communities within the network, the Gephi modularity algorithm was applied, yielding a modularity score of 0.755, indicating a well-defined community structure. This value demonstrates good separation between the nodes within their respective communities, as modularity values close to 1 indicate a strong modular structure, while values near 0 would suggest little or no modularity. The algorithm identified 38 distinct communities (Figure 5), with the first six containing the largest number of nodes: 133, 127, 114, 84, 70, and 46, respectively, for a total of 574 nodes. This concentration suggests that the majority of the network’s structure is organised within a few key communities, where user interactions align with the topics identified through the Reinert Method.

**Figure 6:**
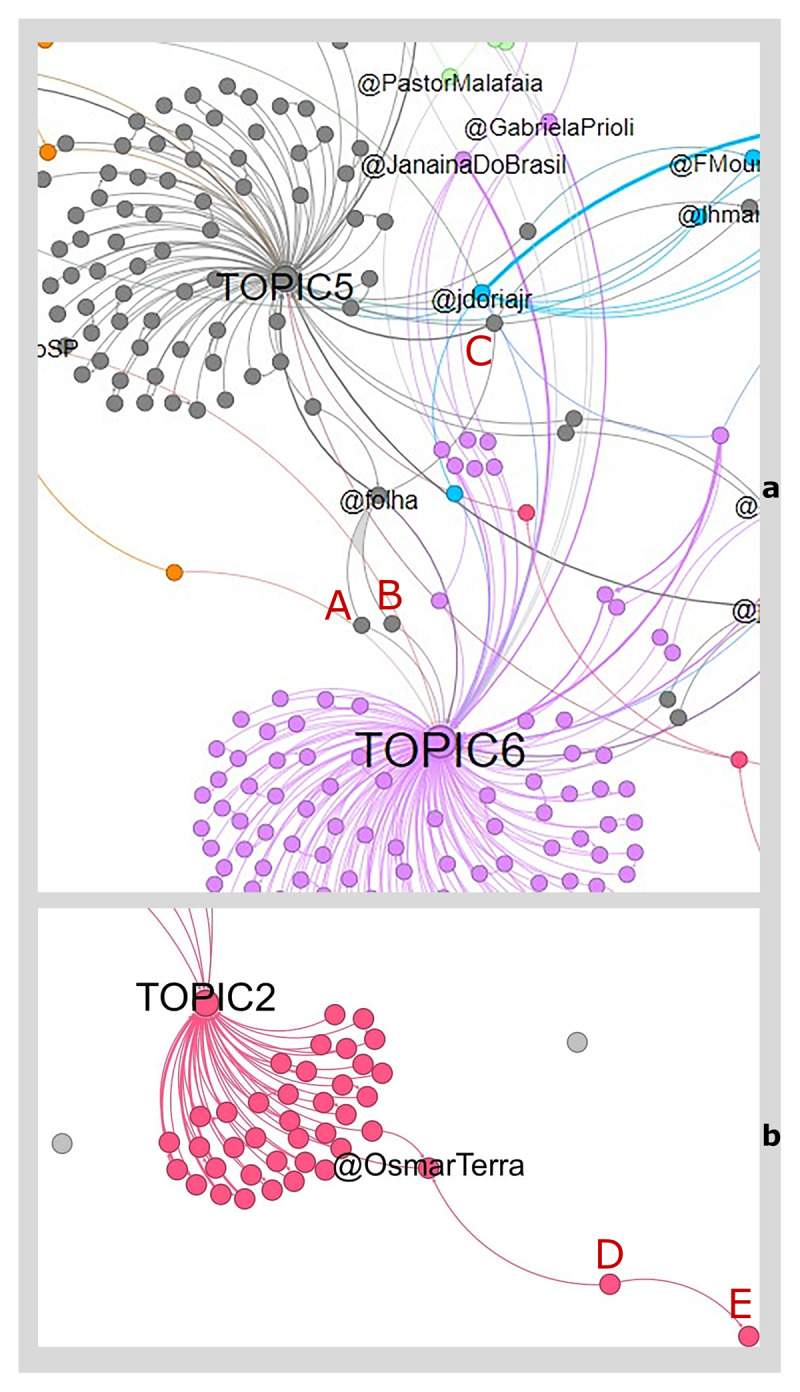
Multiple User-Topic and User-User connections. Note: Zooms of Figure 4; labels A, B, C, D, and E – representing users – have been manually added for clarity of exposition.

This procedure also detects individuals who are associated with a group/community not because of the topic they share with other members, but because of their interactions. For example (Figure 6a), the @folha (an important newspaper from São Paulo) is linked to two topics (i.e., 5, *The actions of the Minister of Health* and 6, *The management of the pandemic*); however, it is statistically associated only to the group that predominantly shares topic 5. When we consider users such as A and B (who share topic 6) and C (who shares more than one topic), we can see that they are all associated with the same community, due to the connection they have with @folha. In other words, their group belonging is not defined by sharing a given topic but by the interaction with another user (i.e., they often mention the newspaper).

Other interesting cases occur with users that are in-between different communities, or that are associated to a group only through another user, as user D and E (Figure 6b). Although it is not directly related to topic 2, the position of the user identified by the letter E in the network may suggest a proximity to this topic and a greater distance from other topics, due to the intermediation carried out by the user identified by the letter D.

## Discussion

This paper proposes an approach rooted in SRT that enables community detection by considering both shared content (operationalized as topics) and individual interactions in communication (in a virtual space, operationalized as tweet mentions). In operational terms, applying both topic detection and bipartite network analysis means considering both individual and topics as nodes and considering both sharing the same topic(s) (sharing the same contents of the representation) and mentioning (interacting) as connections (i.e., arcs).

Moliner’s proposal (2023) provides a procedure for community detection by operationalising a hypothetical situation in which a group exists because its members share the same representational contents. Borrowing tools and perspectives from SNA, particularly bipartite network analysis, nodes are represented by themes and people, and links are represented by having shared the same theme(s) (content of the representation). However, this situation does not consider the presence of other connections beyond the sharing of the same themes.

In our proposal we suggest jointly considering topics and communicative interactions.

As for the content of representation, we detected it through topic detection, in particular, Reinert’s method. In Moliner’s (2023) application, contents are defined by shared words; however, considering only words as proxies for representational contents may work well in a task of free associations but may be limited when considering other sources of textual data that are not merely a list of elicited words but complete sentences. In our opinion, considering the topic instead of a single word, if free associations are not used, offers several advantages: 1) it prevents the inclusion of too many words, some of which may be ‘empty’; 2) it promotes a better interpretation of the results, as a word alone, extracted from a sentence, is conceptually different from a theme. It is worth noting, however, that the procedure we describe and propose here does not consider the connotative aspect of the representation; it only highlights its content. It does not detect whether the formed communities share the same attitude towards a topic or not. For the purposes of the current paper, this is not an issue, because the goal is to identify the content and, whether discussed in agreement or disagreement, the interaction is present and the topic is shared. It would be interesting to further understand how, in operational terms, it is possible to detect and observe the connotative component of the representation, and eventually add also this element to the analysis.

Operationalising interaction is not self-evident, and different options have pros and cons. In the analysis conducted we used the *mention* as a proxy of interaction. This allowed for the identification of direct and explicit communicative interaction. However, the use of mentions as a proxy for interaction does not define a group as meant in SRT by itself. In social media studies, this is referred to as a ‘weak tie’, typical of social media, which is suggested to provide informational support and act as a bridge (Krämer et al. 2014). Nevertheless, using mentions alongside the sharing of the same ideas and thus the same representational content aligns with the definition of communities in the social media literature and that of groups in SRT. Sharing the same representational content helps define the boundaries of a group (and vice versa), and this sharing takes place through communicative exchange, through interaction. In this sense, we refer to reflexive groups (Wagner 1995), as their members actively participate in the construction of shared meaning and refer to a common normative framework, though not in the full sense proposed by Wagner (1995), since there is not necessarily an awareness of belonging. Similarly, in the context of network analysis and social media studies, a group is defined by a higher density of information exchange (Recuero 2009), meaning that there is not only a shared topic but also interaction around this topic.

However, basing it on mentions can easily result in a very dispersed network. In fact, as also in our example, communities are mainly determined by sharing the same representational content and mentions are rare. As an alternative operationalisation of the interaction, we could have generated a network based on retweets; however, this would have resulted in an even more homogeneous network in terms of content. It should be underlined that all these measures of interactions are based on data which are already explicit and available. However, they do not grasp other more subtle forms of interactions, such as reading the previous tweets, and spending more or less time on one message or another. In the analysis of social media, more fine-grained analyses are thus needed to detect (and eventually measure) these practices, which are relevant forms of interaction as well in the sociogenesis and microgenesis of social representations.

We used data from Twitter (X), which are common in SNA, however other types of data could also allow for the operationalisation of different types of interactions. For example, questionnaires could include items that account for the connection with other participants (e.g., the classic sociometric approaches to measure interactions in a classroom). These kinds of measures could also allow to collect longitudinal data, and thus to examine how interactions between individuals and between individuals and topics co–evolve across time.

Finally, putting topics and interaction together, despite the uncertainty regarding the statistical criteria for detecting communities identified by Moliner (2023), this study empirically suggests that the procedure for detecting communities by means of modularity can be effectively useful. On the problem of detecting communities in networks, Blondel et al. (2008) point out that precise formulations of this problem are computationally intractable. However, this does not eliminate the use of these algorithms, which use highly connected nodes in a common cluster as a criterion for forming sub-communities, regardless of the modularity algorithm used (Cherven 2015). Even with the limitations of the data we used, this approach allowed us to identify different communities, and different connections defining groups, mainly based on being linked to a shared topic, but in some cases, being linked to some other individual in the network.

## Conclusions

In this article, we aimed to integrate SNA with topic detection in the study of SRs. We believe that the simultaneous analysis of the twofold relationship *actor-actor* and *actor-content* represents an important advance for the study of SRs, providing a clear visualisation structure for this interaction.

A key contribution of this paper is the proposal to detect communities by considering both shared representational content (topics) and interaction between individuals. This approach aligns with the definition of groups in SRT, where members actively engage in the meaning-making process while defining group boundaries.

We employed basic metrics to interpret the relationships between the nodes of the network, which proved sufficient for the analysis presented. However, we acknowledge that a wide range of other metrics could be explored in future research. For example, metrics like betweenness centrality could be used to identify bridge actors, while PageRank could highlight social influencers or those with greater social capital. The use of these metrics goes beyond the purpose of this paper, they should align with the specific objectives and questions of each investigation, such as understanding the key roles of certain information sources.

Moreover, future studies with longitudinal data could focus on deepening the analysis of group formation and topic sharing over time, enabling researchers to assess the stability and change of these groups in relation to the topics discussed.

Finally, we would like to point out that there are still few studies linking SNA with SRT. We hope this work encourages further research into the potential of SNA for studying SRs. While there is still much to develop in this area, the combination of these two frameworks offers promising avenues for understanding the dynamics of social knowledge and group structures.

## Data Accessibility Statement

The dataset, analysis procedure, and outputs are available at https://osf.io/6ktm9/?view_only=6a7d8b39f7d24ca6b4952c4756fc12f9.

## Footnotes

Twitter (X) has been and is still a source largely used for topic detection and SNA. Indeed, this social media has been studied to identify influential users (Grandjean, 2016), and to understand patterns of information flow within topical networks (Himelboim et al., 2017).

for an extended discussion of network elements refer to the supplementary materials.

The procedure is explained step by step at https://osf.io/6ktm9/?view_only=6a7d8b39f7d24ca6b4952c4756fc12f9 under the “Wizard” component. The datasets used are available at the same link, which is also provided in the supplementary material.

TypeScript code can be accessed via the link https://github.com/bgcorazza/twitter-api.

The total number of users in the network is the sum of users who have written posts and users who have been quoted in the posts. For example, in a post written by user A that mentions user B, there are two users involved, but only user A authored the post. Since some users have written multiple posts, the number of unique users who have authored posts (n = 367) is lower than the total number of posts.
